# Evaluation of new robust silk fibroin hydrogels for posterior scleral reinforcement in rabbits

**DOI:** 10.3389/fbioe.2023.1211688

**Published:** 2023-06-14

**Authors:** Yule Xu, Qiaolin Chen, Zhengzhong Shao, Jiahong Wei, Xuyou Zhu, Ao Rong, Xin Chen, Yusu Ni, Yi Jiang

**Affiliations:** ^1^ Department of Ophthalmology, Tongji Hospital, School of Medicine, Tongji University, Shanghai, China; ^2^ State Key Laboratory of Molecular Engineering of Polymers, Department of Macromolecular Science, Laborarory of Advanced Materials, Fudan University, Shanghai, China; ^3^ Department of Pathology, Tongji Hospital, School of Medicine, Tongji University, Shanghai, China; ^4^ Department of Ophthalmology, Shanghai Xin Shi Jie Eye Hospital, Shanghai, China; ^5^ Otology and Skull Base Surgery Department, Eye and ENT Hospital of Shanghai Medical School, Fudan University, Shanghai, China; ^6^ Key Laboratory of Hearing Medicine of National Health and Family Planning Commission, Shanghai, China; ^7^ Otorhinolaryngology Department of Affiliated Eye and ENT Hospital, State Key Laboratory of Medical Neurobiology, Fudan University, Shanghai, China

**Keywords:** myopia, silk fibroin, biomaterial, posterior scleral reinforcement, biocompatibility

## Abstract

**Background:** Currently, there is no ideal material available for posterior scleral reinforcement (PSR) to prevent the progression of high myopia. In this study, we investigated robust regenerated silk fibroin (RSF) hydrogels as potential grafts for PSR in animal experiments to evaluate their safety and biological reactions.

**Methods:** PSR surgery was performed on the right eye of twenty-eight adult New Zealand white rabbits, with the left eye serving as a self-control. Ten rabbits were observed for 3 months, while 18 rabbits were observed for 6 months. The rabbits were evaluated using intraocular pressure (IOP), anterior segment and fundus photography, A- and B-ultrasound, optical coherence tomography (OCT), histology, and biomechanical tests.

**Results:** No complications such as significant IOP fluctuation, anterior chamber inflammation, vitreous opacity, retinal lesion, infection, or material exposure were observed. Furthermore, no evidence of pathological changes in the optic nerve and retina, or structural abnormalities on OCT, were found. The RSF grafts were appropriately located at the posterior sclera and enclosed in fibrous capsules. The scleral thickness and collagen fiber content of the treated eyes increased after surgery. The ultimate stress of the reinforced sclera increased by 30.7%, and the elastic modulus increased by 33.0% compared to those of the control eyes at 6 months after surgery.

**Conclusion:** Robust RSF hydrogels exhibited good biocompatibility and promoted the formation of fibrous capsules at the posterior sclera *in vivo*. The biomechanical properties of the reinforced sclera were strengthened. These findings suggest that RSF hydrogel is a potential material for PSR.

## 1 Introduction

Myopia is a prevalent ocular disease that has become a global public health issue. The prevalence of myopia in the United States and Europe is around 30%, while in Asian countries, it affects up to 60% of the general population (Saw et al., 2002; McBrien and Gentle, 2003). With increasing severity of myopia, the eyeball continues to elongate. High myopia is commonly defined as a spherical equivalent of ≥6.00 D and is frequently associated with pathologic myopia. Pathological myopia is one of the leading causes of low visual acuity and blindness worldwide, particularly in East Asia, making it a significant medical problem. The pathological changes include posterior scleral staphyloma, retinal choroidal atrophy, choroidal neovascularization, scleral structure and biomechanical streak, and peripheral retinal degeneration (Ohno-Matsui et al., 2016). Posterior staphylomas are a hallmark of high myopia and a significant cause of myopic maculopathy, which can lead to marked visual impairment (Hayashi et al., 2010; Fang et al., 2018).

The sclera is a fibrous and dynamic tissue that makes up the majority of the wall of eyeball. It is mainly composed of superimposed type 1 collagen lamellae with small amounts of elastic fibers embedded in a hydrated matrix of proteoglycans (Keeley et al., 1984; Rada et al., 1997). Thinning of the sclera at the posterior pole of the eye has long been recognized as an essential feature in the development of high myopia (Boote et al., 2020). Research has shown that the total collagen content in the sclera is reduced in high myopia, which can result in the sclera’s deranged metabolism, leading to the weakening of its biomechanical properties. This, in turn, can allow for the continual expansion of the globe in the posterior direction (Gentle et al., 2003; McBrien and Gentle, 2003).

Posterior scleral reinforcement (PSR) involves strengthening the sclera at the posterior pole of the eye to halt the progression of high myopia. The technique was first reported by Shevelev in 1930 and was later modified by Snyder and Thompson (Shevelev, 1930; Snyder and Thompson, 1972; Thompson, 1978). Numerous studies have used various materials in animal experiments or clinical trials to assess the effectiveness and safety of PSR (Jacob-LaBarre et al., 1994; Yan et al., 2010; Huang et al., 2019). Some researchers have been satisfied with the surgical outcomes, while others have been disappointed with the results.

PSR materials are crucial for controlling the progression of high myopia and must exhibit both good biocompatibility and physical properties. PSR surgery has utilized various biological materials such as pericardium, donor sclera, umbilical cord, dura mater, and acellular allograft dermis, as well as non-biological synthetic materials like polyester fibers, polyester mesh sponges, and plasma-modified silicone grafts. Each material has its own advantages and limitations, and challenges to their use include issues such as insufficient availability of donor tissue, the risk of infection, pathogen transmission, and poor durability (Schepens and Acosta, 1991; Jacob-LaBarre et al., 1994; Dereli et al., 2020). Currently, there is no ideal PSR material available to strengthen the weak area of the sclera in the posterior pole and prevent the continuous elongation of the axial length associated with high myopia.

Silk fibroin (SF) is a fibrous protein primarily extracted from the cocoons of *Bombyx mori* silkworms. The amino acid sequence of SF contains repetitive Gly-Ala-Gly-Ala-Gly-Ser repeats, which self-assemble into an antiparallel β-sheet structure. These β-sheets are highly crystalline and essentially crosslink the protein by utilizing strong intra- and intermolecular hydrogen bonds, as well as strong van der Waals forces between stacked β-sheets, thereby improving the mechanical properties of SF (Zhou et al., 2001). Previous research has demonstrated that *in vivo*, only very mild inflammatory responses to SF occur (Wang Y et al., 2008). Regenerated silk fibroin (RSF) has been widely utilized as an excellent biomaterial for various biomedical applications, such as cartilage regeneration (Wang et al., 2006; Hong et al., 2020), artificial blood vessels (Lovett et al., 2007; Zhang et al., 2008; Li et al., 2019), drug delivery scaffolds (Wang X et al., 2008; Maity et al., 2020), ligament reconstruction (Bi et al., 2021; Yan et al., 2021), bone tissue engineering (Hofmann et al., 2007; Gambari et al., 2019), and skin tissue engineering (Bhardwaj et al., 2015). RSF can be fabricated into various forms of materials, including fibers, films, porous sponges, hydrogels, scaffolds, and nanomicrospheres (Kundu et al., 2013; Lu et al., 2015; Yan et al., 2015; Kapoor and Kundu, 2016; Choi et al., 2018). Due to its excellent biocompatibility, remarkable mechanical properties, and controllable biodegradation (Mottaghitalab et al., 2015), RSF holds great promise as a novel biological material for PSR.

In this study, we investigated a new robust RSF hydrogel as a patch for PSR in animal experiments to explore the safety and potential mechanism in preventing the progression of myopia.

## 2 Materials and methods

### 2.1 Fabrication of RSF hydrogels

All chemicals were received and used without further purification. The cocoons of *B. mori* silkworms were purchased from Jiangsu, China. CaCl_2_ and formic acid (FA) were purchased from Sinopharm Chemical Reagent Co., Ltd. The water used in all experiments was deionized using a Millipore purification apparatus (resistivity >18.2 MΩ cm).

The preparation of the robust RSF hydrogels was conducted following the procedure that has been previously reported in our work (Chen et al., 2022). In brief, the degummed silk fibers were dissolved in FA with CaCl_2_ (CaCl_2_/RSF ratio of 0.5) at room temperature for 3 h to make a final RSF concentration of 0.15 g/mL. After defoaming, a transparent homogeneous solution was poured into Teflon molds, which were then immersed in deionized water to form robust RSF hydrogel. The water in which the RSF hydrogel was immersed was frequently replaced with fresh water until the pH of the immersion water was nearly neutral.

All RSF hydrogels were 1 mm thick and cut into 25 mm × 6 mm strips (Supplementary Figure S1). They were sterilized in 75% ethanol at 4°C before implantation.

### 2.2 Animals

A total of twenty-eight adult male New Zealand white rabbits weighing 2.3–2.5 kg were utilized in this study. They were randomly allocated to group 1 (*n* = 10), group 2 (*n* = 10), and group 3 (*n* = 8). Ten rabbits (group 1) were monitored for 3 months, while 18 rabbits (groups 2 and 3) were monitored for 6 months. The right eye was selected as the treated eye, while the left eye served as the contralateral self-control. Prior to surgery, routine eye examinations were conducted, including intraocular pressure (IOP), anterior segment, fundus photography, A-ultrasound, B-ultrasound, and optical coherence tomography (OCT), to establish a baseline and exclude any pre-existing eye diseases. All experimental procedures were carried out in accordance with the Association for Research in Vision and Ophthalmology (ARVO) statement regarding the use of animals in ophthalmic and vision research. The study protocol was reviewed and approved by the Animal Treated Ethics Committee of Tongji Hospital Affiliated to Tongji University, China (authorized number: 2020-DW-006).

### 2.3 Surgical procedures

**FIGURE 1:**
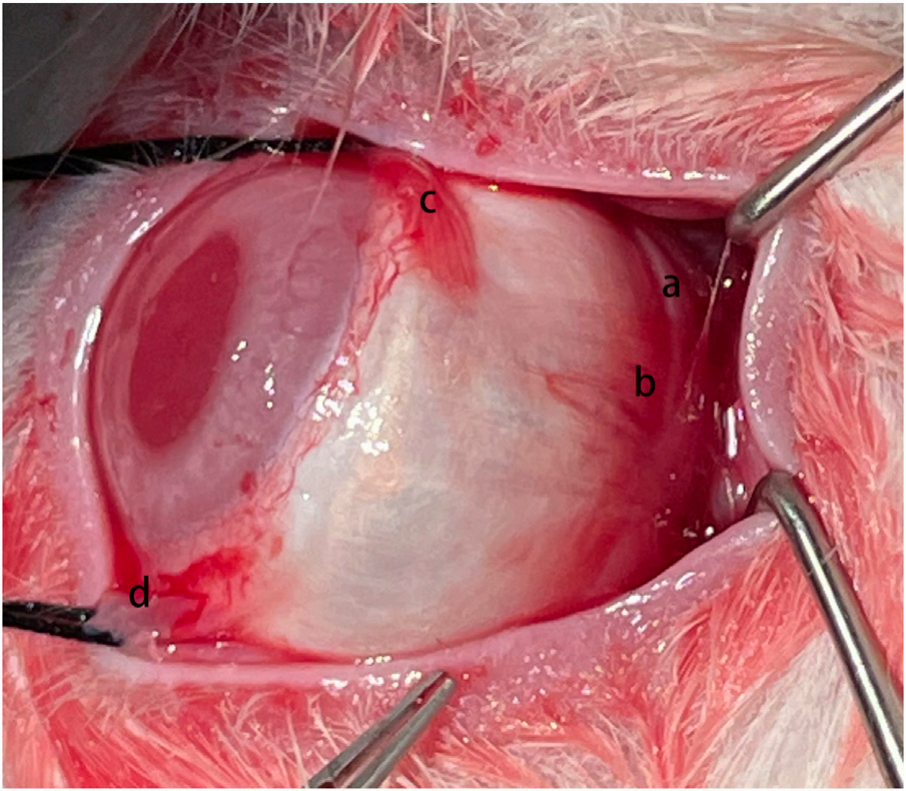
Surgical Procedure. The silk fibroin graft (a) was passed underneath the inferior oblique muscle (b), inferior rectus muscle (c), and lateral rectus muscle (d) sequentially.

The PSR surgery was performed using a single strip, as previously described (Thompson, 1978). The animals were anesthetized by intravenous injection of 3% sodium pentobarbital solution at a dose of 1 mL/kg, along with topical proparacaine hydrochloride (Alcaine, 0.5%, Alcon-Couvreur, Puurs, Belgium). The surgery was conducted by an experienced doctor using an OPMI Lumera T surgical microscope (Carl Zeiss Meditec, Jena, Germany). A 180° conjunctival peritomy was carried out along the corneal limbus at the 4:00–10:00 clockwise position. A radial incision was made at each end of the peritomy, and traction sutures were placed under the inferior and external rectus muscles. With the help of traction sutures and muscle hooks, the strip was sequentially passed beneath the inferior oblique, inferior, and external rectus muscles. One end of the strip was secured to the sclera in the inferior-nasal quadrant using two 10–0 nylon sutures, and the other end was secured to the sclera in the superior temporal quadrant using the same technique. The strip was in good alignment with the outer surface of the posterior pole of the eye globe but was not tightened enough to compress it (Figure 1). Finally, the conjunctival incision was sutured with 10–0 nylon sutures. After surgery, all eyes were treated with levofloxacin eye drops (0.5% Cravit; Santen Pharmaceutical Co., Ltd. Osaka, Japan) four times daily for 1 week.

### 2.4 Postoperative clinical examinations

**FIGURE 2:**
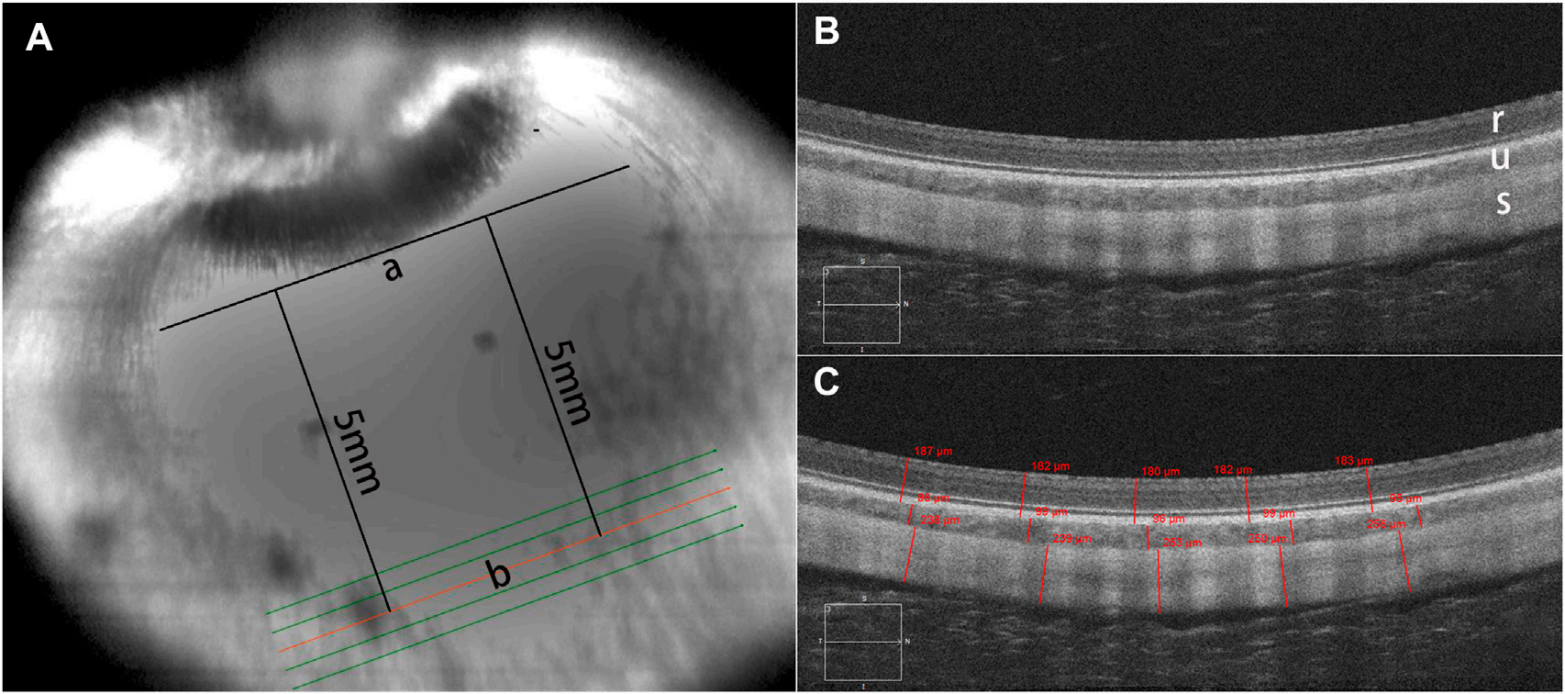
OCT scanning mode. Optical coherence tomography (OCT) was used to examine the microstructure of the retina, choroid, and sclera in the reinforcement area **(A)**. A tangent line (a) was marked at the lower edge of the optic nerve head (ONH) in the infrared (IR) image. The scanning direction was parallel to the tangent line, approximately 5 mm away from it. Enhanced-depth imaging (EDI) images were captured using the HD 5-Line Raster with a spacing of 0.25 mm, consisting of 6 mm parallel lines. There was no structural disorganization in the retinal layers, choroid, and sclera after posterior scleral reinforcement (PSR) surgery **(B)**. A sectional image (b) closest to 5 mm below the tangent line was used for thickness measurement. Five locations of the sectional HD images were averaged for thickness analysis **(C)**. In the images, r represents the retina, u represents the choroid, s represents the sclera.

To assess the safety and biological reactions of the RSF scaffold material, postoperative clinical examinations were conducted at 1 week, 1 month, 3 months, and 6 months using a slit lamp (SL-1800; NIDEX Co., Ltd. Aichi, Japan) and a rebound tonometer (TAO11; Icare Finland Oy, Vantaa, Finland) to record the anterior segment and intraocular pressure (IOP). At 3 and 6 months after the surgery, A-ultrasound (Aviso; Quatel Medical Co., France) was used to measure the length of the ocular axis, B-ultrasound (Aviso; Quatel Medical Co., France) was utilized to detect vitreous changes and the relative position of the RSF scaffold graft material with the posterior sclera, and fundus photography was captured using a fundus camera (TRC-50DX; Topcon Co., Tokyo, Japan) to monitor the dynamic changes in retinal morphology and structure. Spectral domain OCT (Zeiss Cirrus HD-5000; Carl Zeiss Meditec, Dublin, CA) was also performed at the inferior optic nerve head (ONH) location (Figure 2A) to examine the microstructure and thickness of the retina, choroid, and sclera in the reinforcement area. The thicknesses of the retina, choroid, and sclera were measured using the proprietary software of the OCT machine. Five locations of the sectional HD images were averaged for thickness analysis (Figure 2C).

At 3 and 6 months after the surgery, all rabbits were sacrificed, and both eyeballs were immediately enucleated, grossly examined, and photographed. Ten rabbits in group 1 and 2 were randomly divided into subgroups A (*n* = 6), B (*n* = 2), and C (*n* = 2), respectively. Biomechanical testing was performed on eight rabbits in group 3 at 6 months after the surgery.

### 2.5 Histochemistry and immunohistochemistry

In subgroup A (*n* = 6), the eyes were fixed in 10% formalin for 48 h. The eyeballs were sectioned vertically through the central part of the reinforcement material and optic nerve, and then embedded in paraffin for light microscopy. Sections were cut at a thickness of 5 μm. Deparaffinized and rehydrated sections were stained with Hematoxylin & Eosin (HE), Masson’s trichrome, and Verhoeff’s Van Gieson to identify inflammation, vascularity, and formation of fibrous tissue, respectively.

The slides were observed under a light microscope (Eclipse E100; Nikon Co., Ltd. Japan) and the images were transferred into a computer using a digital video camera (DS-U3; Nikon Co., Ltd. Japan). Morphometric analysis was performed on five slides for each sample. Computer-aided analysis with ImageJ software (1.53 k, NIH, United States) was used to quantitatively assess the percentage of collagen and elastic fiber area. All images were digitized to standardize the light intensity of the microscope and condenser height before the quantification process. The arithmetic mean of the percentage measured in the six fields of each slide was used for statistical analysis.

Evaluation of inflammation and vascularization was graded on a scale of 0–4 according to a modified grading system (Kassem et al., 2011; Dereli et al., 2020). The grading guidelines for inflammation and vascularization are listed in Supplementary Table S1.

Transforming growth factor beta (TGF-β) is the primary factor that drives fibrosis. For immunohistochemical staining, serial paraffin sections were deparaffinized and heated in a microwave oven for 15 min for antigen retrieval. After naturally cooling to room temperature and three washes (5 min each) in phosphate-buffered saline (PBS), sections were incubated with 3% hydrogen peroxide in distilled water for 25 min to block endogenous peroxidase, washed three times (5 min each) in PBS, and blocked in 3% BSA for 30 min at room temperature. Subsequently, the sections were incubated overnight with rabbit polyclonal anti-TGF-β1 antibody (GB13028; Servicebio, Wuhan, China) at a 1/100 dilution in a humidified chamber at 4°C. After washing with PBS, the sections were incubated with horseradish peroxidase-conjugated goat anti-rabbit IgG antibody (GB23303; Servicebio, Wuhan, China) at a 1/200 dilution for 50 min at room temperature. The sections were then stained with 3,3′-diaminobenzidine (K5007; DAKO, Denmark) and counterstained with hematoxylin for 3 min. Six randomly selected areas on each slide were evaluated under a light microscope (Eclipse E100; Nikon Co., Ltd., Japan). Photomicrographs were captured using a camera (DS-U3; Nikon Co., Ltd., Japan). ImageJ software was used to quantitatively assess the value of the average optical density (AOD) to estimate TGF-β1 expression.

All images were independently assessed by two researchers (JW and XZ), and a consensus agreement was reached.

### 2.6 Tunel staining

For TUNEL staining, the eyes from subgroup B (*n* = 2) were fixed in 4% paraformaldehyde for at least 24 h, followed by dehydration in 15% sucrose solution at 4°C and then transferred to 30% sucrose solution for further dehydration. The samples were embedded in Tissue-Tek (Sakura, Tokyo, Japan) and sectioned at 8 μm using a cryostat. Apoptotic cells were detected using a commercially available fluorescein (FITC) TUNEL Cell Apoptosis Detection Kit (G1501, Servicebio, Wuhan, China) according to the manufacturer’s instructions. The samples were examined and imaged using a fluorescence microscope (Nikon Eclipse CI; Nikon Co., Ltd. Japan).

### 2.7 Transmission electron microscope

In subgroup C (*n* = 2), the eyes were fixed with 2% glutaraldehyde. Small sections of the wall in the reinforcement area and the optic nerve were cut, dehydrated, and embedded in Embed-812 (Electron Microscopy Sciences, Washington, PA, United States) for transmission electron microscopy (TEM). After polymerization, 60–80 nm thick sections were stained with uranium acetate and lead citrate and examined using an HT7800 transmission electron microscope (Hitachi, Ltd., Tokyo, Japan).

### 2.8 Biomechanical test

To evaluate the biomechanical effect of the RSF graft, stress-strain measurements were performed on the sclera of eight rabbits 6 months postoperatively. The rabbits were euthanized and their whole eyeballs were enucleated. The rabbit sclera adhered to the reinforcement material was harvested using a sharp blade and cut into a 2.5 mm wide and 15 mm long strip for measurement. The tissue adjacent to the strip was carefully removed. Scleral strips were also collected from the corresponding positions of the contralateral eye. The scleral thickness was measured at the center and on both sides of each sample using an electronic digital caliper from Guanglu Measuring Instrument Co., Ltd. in Guilin, China. These strips were clamped vertically at a distance of 8.0 mm between the jaws of an Instron 5943 System, a computer-controlled biomaterial tester from Instron Co., Ltd. in Massachusetts, USA. The strain was linearly increased at a velocity of 2 mm/min until the scleral strip ruptured. Ultimate stress was recorded as the stress on the tissue at the tearing point. The elasticity moduli of all the scleral strips were calculated using the testing system.

### 2.9 Statistical analysis

Data analysis was conducted using SPSS V.26.0 (SPSS Inc., Chicago, IL, United States). Continuous variables were presented as mean ± standard deviation (SD). When multiple measurements were taken from different parts of the same slide or image, the arithmetic mean was used for subsequent statistical analysis. Repeated-measures analysis of variance (ANOVA) was utilized to assess differences in different pre- and postoperative periods. A paired sample *t*-test was used to determine statistical differences between the treated and contralateral control eyes. An independent sample *t*-test was performed to compare data from 3 to 6 months after surgery. The Mann-Whitney U test was used to compare ranked data between groups. A significance level of *p* < 0.05 was considered statistically significant.

## 3 Results

### 3.1 Clinical evaluation

**FIGURE 3:**
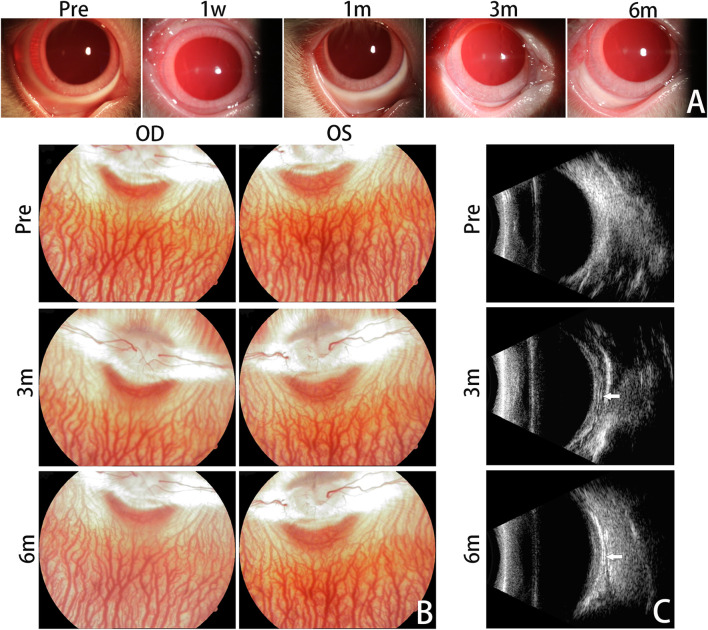
Clinical Evaluation. Anterior segment photographs are presented for the treated eyes before surgery and at 1 week, 1 month, 3 months, and 6 months after the surgery **(A)**. No significant complications emerged within the 6-month follow-up period. Mild conjunctival congestion was observed at 1 week postoperatively and had completely resolved by the first month. Fundus photographs were taken to monitor the dynamic changes of the vitreous body and retina **(B)**. No abnormal fundus changes were observed from 3 months to 6 months postoperatively, compared with pre-operation. Representative B-ultrasound images are displayed for the treated eyes before surgery and at 3 months and 6 months postoperatively **(C)**. The silk fibroin graft could be clearly observed and was characterized by an arcuate band (white arrows). OD, oculus dexter; OS, oculus sinister.

The treated eyes showed no signs of anterior chamber inflammation, with only mild conjunctival congestion observed in the early postoperative period, which completely resolved in all rabbits within the first month (Figure 3A). Corneas were normal and lenses were clear in all rabbits, with no observed vitreous or retinal lesions during the follow-up period (Figure 3B).

B-ultrasound images at 3 and 6 months postoperatively showed that the RSF graft was clearly visible as an arcuate hypo-echo space and high-echo band between the posterior sclera and orbital wall. The RSF graft was appropriately located and adhered well to the posterior sclera (Figure 3C).

Baseline OCT evaluation of retinal morphology showed that the experimental rabbit eyes had normal retinal anatomy. There were no structural disorganization or changes in the retinal layers, choroid, and sclera after PSR surgery (Figure 2B).

### 3.2 Changes of IOP

**FIGURE 4:**
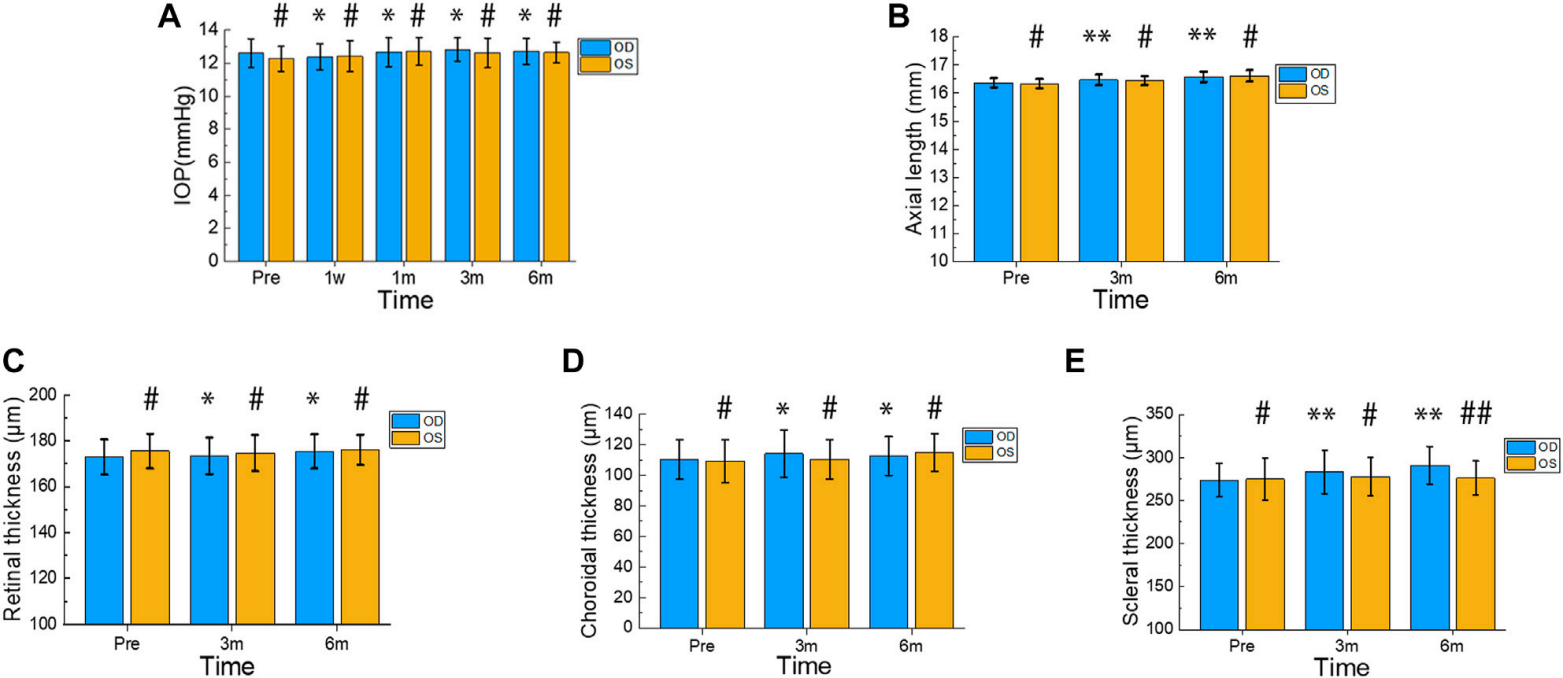
Changes in IOP, Axial Length, Retinal Thickness, Choroidal Thickness, and Scleral Thickness during Postoperative 6 Months. Intraocular pressure (IOP) was measured in the treated and contralateral eyes before surgery and at 1 week, 1 month, 3 months, and 6 months after surgery **(A)**. Axial length was assessed in the treated and contralateral eyes before surgery and at 3 months and 6 months after surgery **(B)**. The thickness of the retina, choroid, and sclera was measured in the treated and contralateral eyes before surgery as well as 3 and 6 months after surgery **(C–E)**. In the treated eyes group, differences in IOP before and after surgery were indicated by * or **. Differences in IOP between the treated and contralateral eyes at each time point were indicated by # or ##. Error bars represent the standard deviation. #*p* > 0.05, *p > 0.05, ##*p* < 0.05, **p < 0.05.

The changes in IOP after surgery are presented in Figure 4A. Compared to the preoperative IOP (12.61 ± 0.88 mmHg), there were no significant differences in the treated eyes at postoperative 1 week, 1 month, 3 months, and 6 months (12.38 ± 0.80 mmHg, 12.68 ± 0.87 mmHg, 12.84 ± 0.71 mmHg, and 12.72 ± 0.78 mmHg; *p* = 0.390, *p* = 0.794, *p* = 0.343, and *p* = 0.591, respectively). Moreover, there was no significant difference in IOP between the treatment group and the contralateral group at the same time points (12.28 ± 0.76 mmHg, 12.43 ± 0.94 mmHg, 12.72 ± 0.84 mmHg, 12.63 ± 0.91 mmHg, and 12.66 ± 0.66 mmHg; *p* = 0.231, *p* = 0.880, *p* = 0.892, *p* = 0.433, and *p* = 0.798, respectively).

### 3.3 Changes of AL

Figure 4B displays the changes in axial length. The treated eyes showed significant differences (*p* = 0.002 < 0.01, *p* < 0.001) in comparison to their preoperative axial length (16.37 ± 0.39 mm), with measurements of 16.47 ± 0.19 mm and 16.58 ± 0.87 mm at postoperative 3 months and 6 months, respectively. Moreover, no significant difference was observed in axial length between the treatment and contralateral groups at the same time point (16.34 ± 0.16 mm, 16.44 ± 0.16 mm, 16.61 ± 0.2 mm; *p* = 0.620, *p* = 0.640, *p* = 0.660). At 6 months after surgery, both the treated and control eyes exhibited an increase in axial length by 0.21 mm and 0.27 mm, respectively.

### 3.4 Changes about thickness of retina, choroid, and sclera

Figures 4C, D illustrate the changes in retinal and choroidal thickness, respectively. The treated eyes exhibited relatively gentle fluctuations in retinal and choroidal thickness during the postoperative period. Compared to the preoperative retinal thickness (172.89 ± 7.69 µm), the treated eyes showed no significant differences (173.50 ± 7.94 µm, 175.39 ± 7.49 µm; *p* = 0.628, *p* = 0.062) at 3 and 6 months postoperatively. Furthermore, there were no significant differences in retinal thickness between the treated and contralateral eyes at the same time points (175.56 ± 7.52 µm, 174.67 ± 7.90 µm, 176.06 ± 6.65 µm; *p* = 0.300; *p* = 0.661, *p* = 0.799). The preoperative choroidal thickness was 110.39 ± 12.87 µm, and the postoperative choroidal thickness at 3 and 6 months was 114.17 ± 15.26 µm and 112.78 ± 12.77 µm, respectively. There were no significant differences between pre- and postoperative values (*p* = 0.218 and *p* = 0.426, respectively). Additionally, the treated eyes’ choroidal thickness did not differ significantly from that of the contralateral eyes at the same time points (109.28 ± 13.91 µm, 110.56 ± 12.99 µm, 115 ± 12.26 µm; *p* = 0.805, *p* = 0.450, *p* = 0.598).

Figure 4E shows the changes in scleral thickness over time. Compared to the preoperative scleral thickness (273.67 ± 19.46 µm), the treated eyes showed a significant increase (283.33 ± 25.25 µm, 290.94 ± 21.91 µm; *p* = 0.004, *p* < 0.001) at 3 and 6 months postoperatively. Six months after surgery, the treated eyes’ scleral thickness increased by 17.27 µm. There were no significant differences in scleral thickness between the treated and contralateral eyes at the preoperative and 3-month time points (275 ± 24.36 µm, 277.94 ± 22.67 µm; *p* = 0.857, *p* = 0.505). However, the treated eyes’ scleral thickness showed a statistically significant difference compared to the contralateral eyes’ at 6 months postoperatively (276.17 ± 19.66 µm; *p* = 0.041).

### 3.5 Gross examination

**FIGURE 5:**
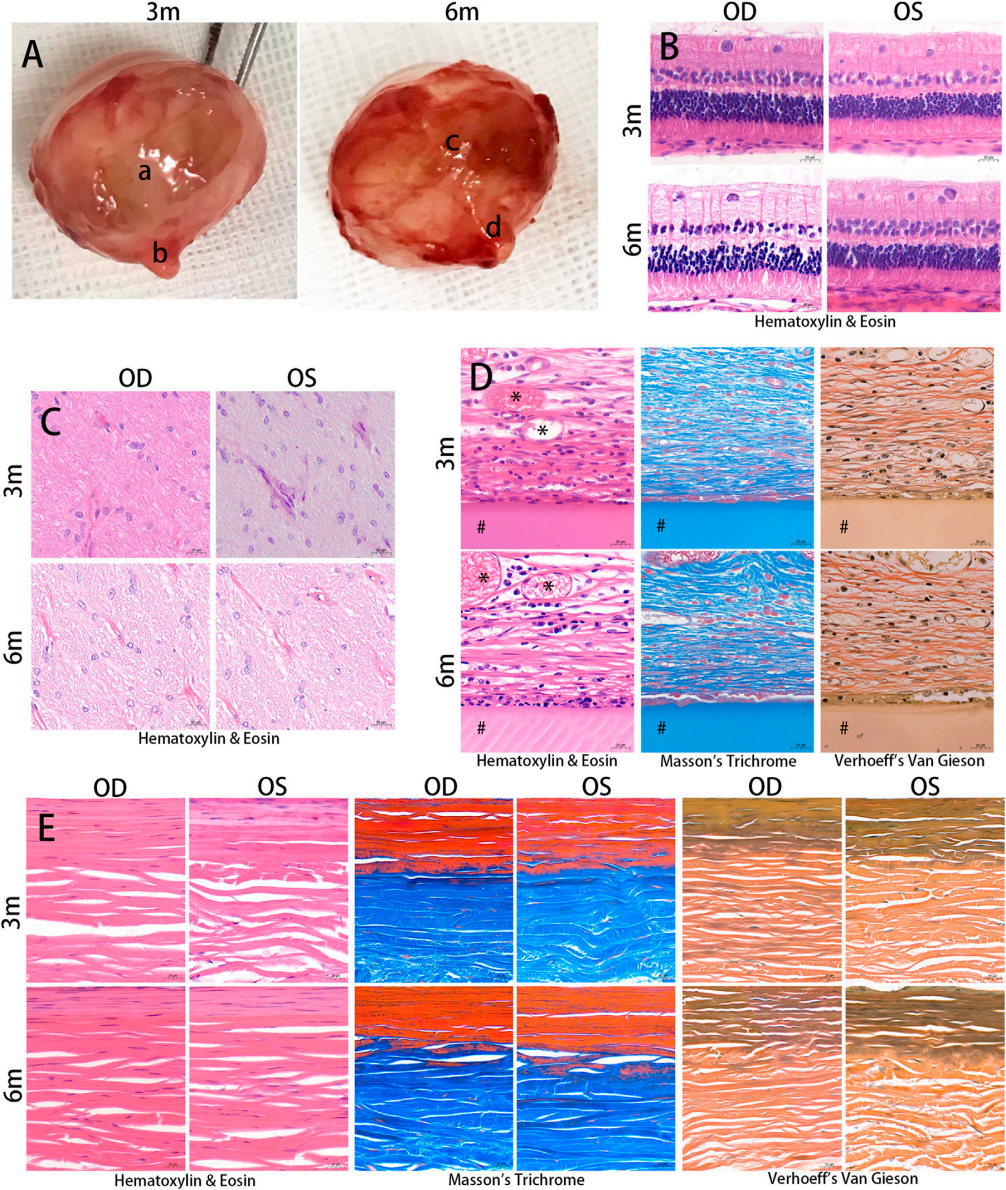
Histological examination. The eyeballs of the animals were enucleated and photographed at 3 and 6 months after the surgery **(A)**. Silk fibroin grafts were enclosed in a fibrous capsule (a, c). The optic nerves appeared normal in the treated eyes (b, d). The samples were embedded in paraffin and stained with Hematoxylin & Eosin, Masson’s trichrome, and Verhoeff’s Van Gieson. Hematoxylin & Eosin images showed no structural abnormalities or inflammatory cells in the retina and optic nerve in the treated and contralateral eyes at 3 months and 6 months postoperatively **(B, C)**. The silk fibroin graft (#) was surrounded by a fibrous capsule containing collagen and a number of inflammatory cells **(D)**. Vascular ingrowth (*) was evident in the fibrous capsule. The diameter of the scleral collagen fibrils varied in thickness **(E)**. OD, oculus dexter; OS, oculus sinister. Scale bar = 20 µm.

Upon gross examination of the enucleated eye globes, it was observed that all grafts were surrounded by a fibrous capsule that could not be easily separated from the rabbit eyeball (Figure 5A). None of the grafts were displaced from the reinforced area at any point after PSR. When the fibrous capsule was incised, the RSF material remained intact for up to 3 months after surgery. However, after 6 months, the materials showed partial decomposition. The posterior edge of the RSF graft was located approximately 4 mm from the optic nerve, while the optic nerves appeared normal in all specimens. Both treated and untreated eyes appeared to be of similar size and shape.

### 3.6 HE, Masson’s trichrome and Verhoeff’s Van Gieson staining

Following implantation, histological examination of the HE staining revealed no evidence of pathological changes or structural abnormalities in the retina, choroid, and optic nerve (Figures 5B, C). The RSF graft was enclosed in a fibrous capsule containing collagen (Figure 5D). The RSF graft elicited a number of inflammatory cells, including plasma cells, lymphocytes, macrophages, neutrophils, and fibroblasts, which were observed around the graft. Vascular ingrowth was also evident at the graft periphery. The degree of inflammation and vascularization of the fibrous capsules surrounding the RSF graft is presented in Table 1. The inflammatory reaction at 3 months was slightly more evident than at 6 months, with a score of 1.67 ± 0.65 decreasing to 1.50 ± 0.52. However, there was no statistically significant difference between the two time points (*p* = 0.423). Mildly increased vascularity was observed in all treated eyes, while no inflammation or vascularization was observed in the contralateral eye.

**TABLE 1:** Inflammation and vascularization of the fibrous capsule around silk fibroin graft.

Animal no.	Inflammation score		Vascularity score	
	Treated eye	Contralateral eye	Treated eye	Contralateral eye
1^a^	2	0	3	0
2^a^	1	0	3	0
3^a^	2	0	3	0
4^a^	3	0	3	0
5^a^	1	0	3	0
6^a^	2	0	3	0
7^b^	2	0	3	0
8^b^	1	0	3	0
9^b^	1	0	3	0
10^b^	2	0	3	0
11^b^	2	0	3	0
12^b^	1	0	3	0

^a^ Rabbit executed at month 3.

^b^ Rabbit killed at month 6.

The diameter and lamellae of the scleral collagen fibrils exhibited varying thicknesses (Figure 5E). Changes in the density of scleral collagen, as determined by Masson’s trichrome and Verhoeff’s Van Gieson staining, are presented in Table 2. At month 3, the percentage of collagen fiber area in the treated eyes was significantly higher than in the contralateral eyes, as measured by Masson’s trichrome staining or Verhoeff’s Van Gieson staining (51.57% ± 1.76% and 47.60% ± 1.81%, respectively, compared to 42.84% ± 2.19% and 42.25% ± 1.85% in the contralateral eyes; *p* < 0.001 and *p* = 0.002, respectively). The treated eyes also showed a significant difference in collagen fiber area compared to the contralateral eyes at month 6 (49.74% ± 4.12% and 48.17% ± 2.22%, respectively, compared to 44.37% ± 1.27% and 42.61% ± 1.29% in the contralateral eyes; *p* = 0.015 and *p* = 0.004, respectively). No significant changes were observed in the percentage of collagen fiber area with Masson’s trichrome staining or Verhoeff’s Van Gieson staining in the treated eyes between postoperative months 3 and 6 (*p* = 0.340 and *p* = 0.640, respectively).

**TABLE 2:** Collagen and elastic fiber level in the sclera tissue.

Fiber area (%)	Treated eyes (*n* = 6)	Contralateral eyes (*n* = 6)	*p*-Value
Collagen fiber area at month 3			
With Masson’s trichrome staining			
Mean ± SD	51.57 ± 1.76	42.84 ± 2.19	<0.001^a^
Collagen fiber area at month 6			
With Masson’s trichrome staining			
Mean ± SD	49.74 ± 4.12	44.37 ± 1.27	0.015^a^
*p*-value	0.340^b^	0.167^b^	
Collagen fiber area at month 3			
With Verhoeff’s van gieson staining			
Mean ± SD	47.60 ± 1.81	42.25 ± 1.85	0.002^a^
Collagen fiber area at month 6			
With Verhoeff’s van gieson staining			
Mean ± SD	48.17 ± 2.22	42.61 ± 1.29	0.004^a^
*p*-value	0.640^b^	0.701^b^	
Elastic fiber area at month 3			
With Verhoeff’s van gieson staining			
Mean ± SD	25.46 ± 1.00	25.24 ± 3.35	0.892^a^
Elastic fiber area at month 6			
With Verhoeff’s van gieson staining			
Mean ± SD	25.34 ± 1.27	24.40 ± 2.31	0.441^a^
*p*-value	0.858^b^	0.623^b^	

^a^ Paired-samples *t*-test.

^b^ Independent *t*-test.

The scleral elastic fibers are primarily located in the inner sclera as thin, long fiber networks. Changes in the density of scleral elastic fibers with Verhoeff’s Van Gieson staining are presented in Table 2. In terms of the percentage of scleral elastic fiber area, no significant differences were observed between 3 and 6 months in both the treated and contralateral groups (*p* = 0.858 and *p* = 0.623, respectively). Similar results were obtained between the treated and contralateral eyes at 3 and 6 months postoperatively (*p* = 0.892 and *p* = 0.441, respectively).

Table 3 displays the changes in collagen fiber density of the fibrous capsule around the RSF graft with Masson’s trichrome and Verhoeff’s Van Gieson staining. At postoperative 6 months, a significant increase in the percentage of collagen fiber area with Masson’s trichrome staining was observed in the treated eyes compared to postoperative 3 months (*p* < 0.001). Similar results were found in eyes stained with Verhoeff’s Van Gieson stain (*p* < 0.001).

**TABLE 3:** Collagen fiber level of the fibrous capsule around silk fibroin graft.

Collagen fiber level	3 months (*n* = 6)	6 months (*n* = 6)	*p*-Value
Collagen fiber area (%)			
With Masson’s trichrome staining			
Mean ± SD	36.77 ± 1.61	46.91 ± 3.86	<0.001^a^
With EVG staining			
Mean ± SD	39.20 ± 1.88	44.76 ± 1.78	<0.001^a^

^a^ Independent *t*-test.

### 3.7 Over-expression of TGF-β1 detected through immunohistochemistry

**FIGURE 6:**
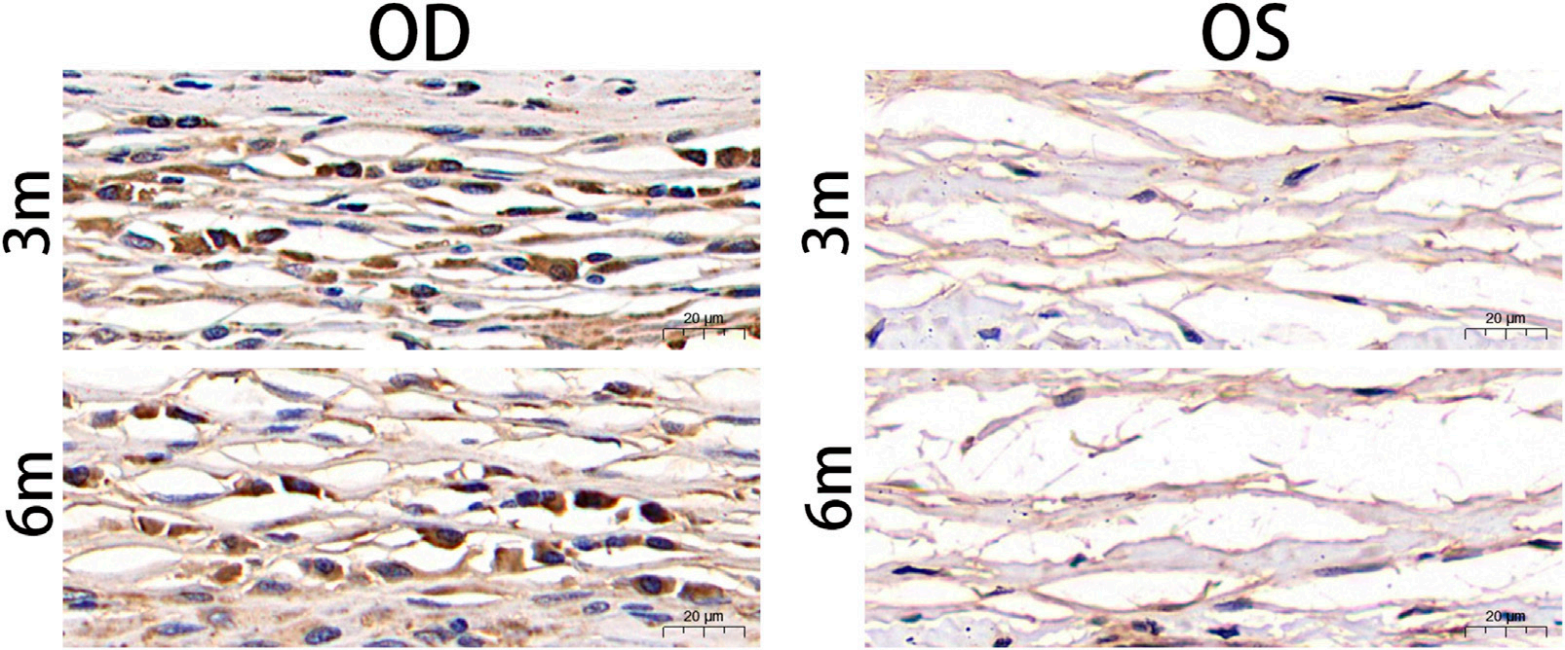
Postoperative photographs of the fibrous capsule around silk fibroin graft based on anti-TGF-β1 antibody immunohistochemical staining. TGF-β1 was significantly upregulated in the treated eyes 3 months and 6 months after surgery. TGF-β, transforming growth factor beta; OD, oculus dexter; OS, oculus sinister. Scale bar = 20 µm.

To investigate the expression of TGF-β1 in the fibrous capsule surrounding the RSF graft, we performed immunohistochemical staining of paraffin sections of the eyeballs at 3 and 6 months postoperatively. The results of the immunohistochemical staining for TGF-β1 are shown in Figure 6, which revealed a significant upregulation of TGF-β1 expression in the treated groups. The AOD value was 0.219 ± 0.012 at 3 months and 0.221 ± 0.033 at 6 months in the treated eyes, as shown in Table 4. However, there was no statistically significant difference between the two time points (*p* = 0.857).

**TABLE 4:** Average optical density (AOD) of fibrous capsule to estimate the transforming growth factor beta1 (TGFβ1) expression.

Time	3 months (*n* = 6)	6months (*n* = 6)	*p*-Value
AOD			
Mean ± SD	0.219 ± 0.012	0.221 ± 0.033	0.857^a^

^a^ Independent *t*-test.

### 3.8 Apoptotic detection of retina

**FIGURE 7:**
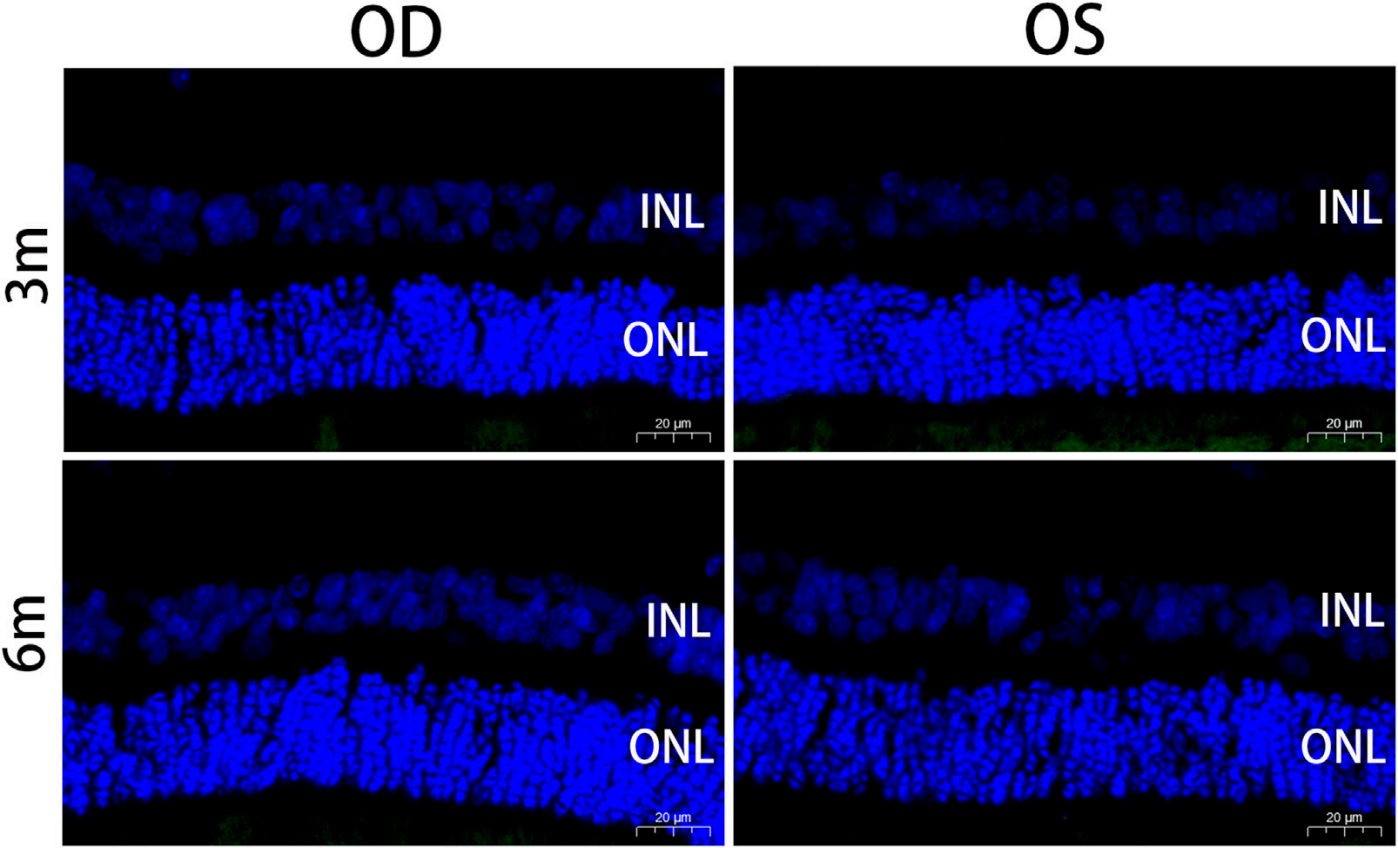
Apoptotic nuclei detected by TUNEL staining in the retina at 3 months and 6 months postoperatively. Positive apoptotic cells were labeled with FITC (green). Sections were counterstained with DAPI (blue). No cellular apoptosis was observed in the INL and ONL of the retina in the treated and contralateral eyes. OD, oculus dexter; OS, oculus sinister; INL, inner nuclear layer; ONL, outer nuclear layer. Scale bar = 20 µm.

The TUNEL assay is a method used to detect fragmented DNA in apoptotic cells. In this study, nuclei were stained blue with DAPI, while apoptotic cells were labeled green with FITC. The absence of cellular apoptosis, as indicated by negative TUNEL results, suggests that there was no significant programmed cell death occurring in either the outer or inner nuclear layers of the retina in both the experimental and control eyes (Figure 7).

### 3.9 Ultrastructural TEM analyses of retina and optic nerve

**FIGURE 8:**
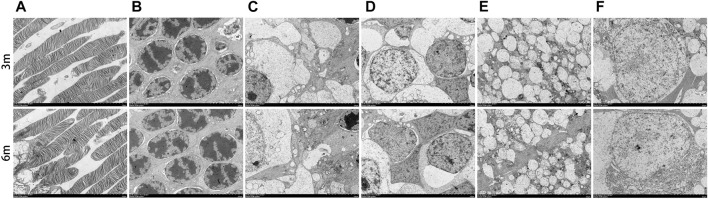
Ultrastructural transmission electron microscopy analyses of retina at 3 months and 6 months postoperatively. No signs of necrosis or apoptosis (including organelle swelling, nuclear shrinkage or condensation, and vacuolization) were found in all retinal layers of the treated eyes **(A–F)**. **(A)**, photoreceptor layer; **(B)**, outer nuclear layer; **(C)**, inner plexiform layer; **(D)**, inner nuclear layer; **(E)**, outer plexiform layer; **(F)**, retinal ganglion cell. Scale bar = 5 µm.

**FIGURE 9:**
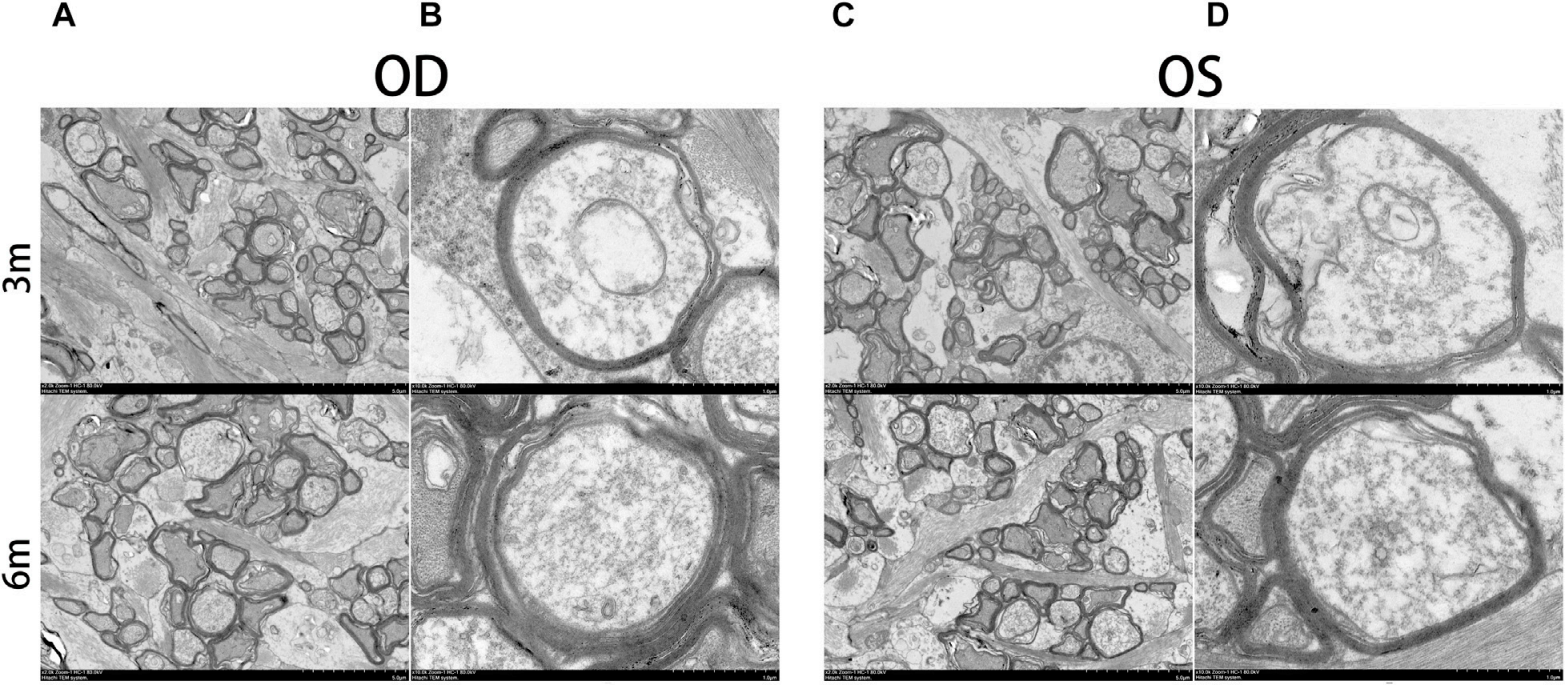
Ultrastructural transmission electron microscopy analyses of optic nerve at 3 months and 6 months postoperatively. No signs of disruption of axon fascicles, degeneration with axon swelling, lower axon numbers, and increases in connective tissue septa were found in the optic nerve of the treated and contralateral eyes. OD, oculus dexter; OS, oculus sinister. Scale bar = 5 µm **(A,C)**. Scale bar = 1 µm **(B,D)**.

The morphology of the retina and optic nerve was further analyzed using transmission electron microscopy (TEM). Figure 8 displays TEM images of the retina at 3 and 6 months after surgery, revealing no observable changes in any of the retinal layers of the treated eyes, including organelle swelling, nuclear shrinkage or condensation, and vacuolization (Figures 8A–F). In Figure 9, TEM images of the optic nerve at 3 and 6 months after surgery show that the myelin sheaths of the optic nerve were normal. Additionally, there were no signs of disruption of axon fascicles, degeneration with axon swelling, lower axon numbers, or increased connective tissue septa in either the treated or contralateral groups.

### 3.10 Biomechanical evaluation of reinforced sclera

The biomechanical parameters of the reinforced sclera 6 months after surgery are presented in Table 5. The ultimate stress was 4.04 ± 1.45 MPa and 3.09 ± 1.00 MPa in the treated and control groups, respectively. There was a significant difference in ultimate stress between the two groups (*p* = 0.023), with a 30.7% increase in the treated group compared to the controls. The elastic modulus of the reinforced sclera was 19.00 ± 7.01 MPa in the treated group and 14.29 ± 5.27 MPa in the control group. Similarly, there was a significant difference in the elastic modulus between the two groups (*p* = 0.035), with a 33.0% increase in the treated group compared to the controls. These findings indicate that reinforced sclera has greater biomechanical stiffness than untreated eyes.

**TABLE 5:** Biomechanical parameters of the sclera at month 6.

Biomechanical parameter	Treated eyes (*n* = 8)	Contralateral eyes (*n* = 8)	*p*-Value
Ultimate stress (MPa)			
Mean ± SD	4.04 ± 1.45	3.09 ± 1.00	0.023^a^
Elasticity modulus (MPa)			
Mean ± SD	19.00 ± 7.01	14.29 ± 5.27	0.035^a^

^a^ Paired-samples *t*-test.

## 4 Discussion

SF is an attractive material for various biomedical applications due to its low immunogenicity, slow degradation profile, resistance to enzymatic cleavage, robust mechanical properties, and ease of processing into different forms (Minoura et al., 1990; Horan et al., 2005; Meinel et al., 2005; Farokhi et al., 2018). In our previous studies, we have confirmed that SF has high biocompatibility because of its unique structure (Ni et al., 2014; Ni et al., 2019). RSF hydrogels are three-dimensional polymer networks with a high water content that are typically formed by physical or chemical cross-linking (Wang and Zhang, 2015). However, traditional RSF hydrogels have a simple network structure, which limits their adaptability to complex environments. In recent years, functional RSF hydrogels with properties such as injectability, self-healing, adhesiveness, environmental responsiveness, electrical conductivity, and 3D printability have attracted researchers’ attention (Zheng and Zuo, 2021). However, their applications are still limited due to their low mechanical strength. In this study, we applied a novel and robust RSF hydrogel and evaluated it as a patch for PSR in normal adult New Zealand white rabbits. In our previous work (Chen et al., 2022), the characterization results of the RSF hydrogel (including mechanical and cytotoxicity properties) have been reported in detail. The mechanical properties can be adjusted by varying the concentration of RSF solution. When the RSF concentration increased from 0.075 to 0.15 g/mL, the compressive modulus of the hydrogel increased from 0.53 to 2.31 MPa, elastic modulus increased from 0.84 to 2.00 MPa, and the tensile strength increased from 0.23 to 0.54 MPa. In addition, the RSF hydrogel showed good biocompatibility by different testing methods, including incubation L929 cells with the hydrogel extracts, culturing L929 cells on the hydrogels surface, and implantation of the hydrogel into subcutaneous tissue of SD rats.

The study indicated that over a 6-month period, RSF hydrogels demonstrated good tolerance and biocompatibility when tested in rabbits. All implanted eyes remained healthy, and no intraocular or periorbital complications, such as infection or material exposure, were observed. Mild conjunctival congestion was observed early after the operation, which completely disappeared in the first month of the study. Additionally, there was no evidence of pathological changes in the optic nerve or retina, and no structural abnormalities were observed in any layer of the retina on OCT. There were no significant intraocular pressure fluctuations during the postoperative observational period. The TUNEL assay was used to detect the DNA fragmentation characteristics of apoptotic cells (Gavrieli et al., 1992), and TUNEL-positive cells were not detected in the retinal sections of the treated eyes at 3 and 6 months postoperatively.

Despite the good biocompatibility of SF, it is still a heterogeneous protein in rabbits and may cause minimal foreign-body reactions. The development of a fibrous capsule around the RSF graft is a biological response to foreign proteins that cannot be eliminated, which isolates the foreign protein from the biological environment to minimize adverse effects. The fibrous capsules that surround long-term implants are typically characterized by the presence of macrophages, giant cells, lymphocytes, fibroblasts, a dense matrix of collagen, and fibrous tissue (Coleman et al., 1974). In our study, the RSF hydrogels were completely fixed inside the fibrovascular capsule, as demonstrated by B-ultrasound, gross, and histological examinations. The RSF graft was firmly attached to the posterior sclera over a wide area by the fibrovascular tissue, resulting in the formation of a sclera-fibrous tissue-material complex. The goal of scleral reinforcement surgery is to prevent global elongation, and the newly formed sclera-fibrous tissue-material complex may have superior biomechanical properties that can block the continuous elongation of the axial length and maintain a stable refractive status.

In our study, we observed infiltration of various inflammatory cells, including plasma cells, lymphocytes, macrophages, neutrophils, and fibroblasts, in the tissue around the RSF graft. The cell-mediated immune response to RSF can result in the synthesis of collagen lamellae, elastic fibers, and the extracellular matrix, leading to the formation of fiber wrapping around the graft. Macrophages and lymphocytes play important regulatory roles under fibrotic conditions by releasing cytokines. TGF-β is a key cytokine that is essential for inducing fibrosis (Biernacka et al., 2011). The immunohistochemistry results of this study showed higher expression levels of TGF-β1 at 3 and 6 months. Although all three isoforms (TGF-β1, 2, and 3) are expressed in fibrotic tissues, the development of tissue fibrosis is primarily attributed to TGF-β1 (Ask et al., 2008). Once TGF-β1 is activated, it promotes fibroblast-to-myofibroblast transition and regulates extracellular matrix remodeling.

The biomechanical properties of the reinforced sclera were found to be enhanced in this study. The ultimate stress of the reinforced sclera increased by 30.7%, and the elastic modulus increased by 33.0% compared to the control eyes. The treated eyes had a significantly higher scleral collagen fiber content than the contralateral eyes at 3 and 6 months after surgery. Additionally, the scleral thickness of the treated eyes increased by 17.27 µm 6 months after surgery. Collagen fiber content and scleral thickness are crucial factors affecting the biomechanical properties of the sclera (Rada et al., 2006). Therefore, increasing collagen content and scleral thickness could potentially enhance the ultimate stress and elastic modulus of the sclera.

The degradation behavior of SF biomaterials is a crucial factor for their *in vivo* medical applications. A critical issue faced by researchers is whether the effect of PSR can be maintained for a longer time. Degradation of the implanted material after PSR surgery can result in the loss of the reinforcement effect (Xue et al., 2014). An ideal implant material should have a degradation rate that matches the healing or regeneration process at the implantation site. According to the United States Pharmacopeia’s definition, silk is classified as a non-degradable biomaterial. However, silk is degradable and slowly absorbed *in vivo* over a long period. Different forms of SF may have highly variable degradation rates depending on factors such as structural and morphological features of the biomaterial, processing conditions, and characteristics of the biological environment at the implant site (Cao and Wang, 2009). Enzymes, especially proteolytic enzymes, play a significant role in the degradation of SF. B-mode ultrasound, computed tomography (CT), HE staining, and scanning electron microscopy are often used to evaluate the biodegradability of SF materials (Lu et al., 2015; Campos et al., 2020; Cai et al., 2021). In this study, although the material had partially decomposed at 6 months after surgery, it still acted as a graft for promoting cell migration and inducing the formation of extracellular matrix on the surface of the sclera. The reinforcement effect provided by the RSF graft was maintained for at least 6 months. Due to the slow degradation of SF, further investigation is necessary to determine the long-term biological reaction of PSR with RSF grafts.

A limitation of our study was that we did not use animal models of myopia to verify the effectiveness of RSF. Animal models of myopia have been established using guinea pigs, chicks, and mice, but their small eyeballs make them unsuitable for PSR. Therefore, rabbits were the most suitable animals for our study. In future studies, we plan to use rabbit models of myopia to evaluate the efficacy of RSF grafts in PSR. Additionally, our study did not include an examination of diopters and electrophysiological functions.

## 5 Conclusion

Our study has revealed that the RSF hydrogel exhibits excellent biocompatibility and is safe for all layers of the retina and optic nerve. The biomechanical properties of the reinforced sclera were improved, likely due to an increase in scleral collagen content and thickness. Additionally, the RSF hydrogel also facilitated the formation of a fibrous capsule around it. The newly formed sclera-fibrous tissue-material complex may possess superior biomechanical properties that can prevent the continuous elongation of the axial length and maintain a stable refractive status. Given its impressive biocompatibility and effectiveness, this novel robust RSF hydrogel holds great promise as a potential material for PSR surgery.

## Data Availability

The original contributions presented in the study are included in the article/Supplementary Material, further inquiries can be directed to the corresponding authors.

## References

[B1] Ask K. Bonniaud P. Maass K. Eickelberg O. Margetts P. J. Warburton D. (2008). Progressive pulmonary fibrosis is mediated by TGF-β isoform 1 but not TGF-β3. Int. J. Biochem. Cell Biol. 40 (3), 484–495. 10.1016/j.biocel.2007.08.016 17931953 PMC2350199

[B2] Bhardwaj N. Sow W. T. Devi D. Ng K. W. Mandal B. B. Cho N.-J. (2015). Silk fibroin–keratin based 3D scaffolds as a dermal substitute for skin tissue engineering. Integr. Biol. 7 (1), 53–63. 10.1039/c4ib00208c 25372050

[B3] Bi F. Chen Y. Liu J. Hu W. Tian K. (2021). Bone mesenchymal stem cells contribute to ligament regeneration and graft-bone healing after anterior cruciate ligament reconstruction with silk-collagen scaffold. Stem Cells Int. 2021, 1–11. 10.1155/2021/6697969 PMC8088362 33981343

[B4] Biernacka A. Dobaczewski M. Frangogiannis N. G. (2011). TGF-β signaling in fibrosis. Growth factors. 29 (5), 196–202. 10.3109/08977194.2011.595714 21740331 PMC4408550

[B5] Boote C. Sigal I. A. Grytz R. Hua Y. Nguyen T. D. Girard M. J. A. (2020). Scleral structure and biomechanics. Prog. Retin. Eye Res. 74, 100773. 10.1016/j.preteyeres.2019.100773 31412277 PMC7187923

[B6] Cai L. Gao N. Sun T. Bi K. Chen X. Zhao X. (2021). Application of an ultrasound semi-quantitative assessment in the degradation of silk fibroin scaffolds *in vivo* . Biomed. Eng. Online 20 (1), 48. 10.1186/s12938-021-00887-3 34006299 PMC8130099

[B7] Campos F. Bonhome-Espinosa A. B. Chato-Astrain J. Sánchez-Porras D. García-García Ó. D. Carmona R. (2020). Evaluation of fibrin-agarose tissue-like hydrogels biocompatibility for tissue engineering applications. Front. Bioeng. Biotechnol. 8, 596. 10.3389/fbioe.2020.00596 32612984 PMC7308535

[B8] Cao Y. Wang B. (2009). Biodegradation of silk biomaterials. Int. J. Mol. Sci. 10 (4), 1514–1524. 10.3390/ijms10041514 19468322 PMC2680630

[B9] Chen L. Sun L. Y. Yao J. R. Zhao B. J. Shao Z. Z. Chen X. (2022). Robust silk protein hydrogels made by a facile one-step method and their multiple applications. ACS Appl. Bio Mat. 5, 3086–3094. 10.1021/acsabm.2c00354 35608071

[B10] Choi J. H. Kim D. K. Song J. E. Oliveira J. M. Reis R. L. Khang G. (2018). Silk fibroin-based scaffold for bone tissue engineering. Adv. Exp. Med. Biol. 1077, 371–387. 10.1007/978-981-13-0947-2_20 30357699

[B11] Coleman D. L. King R. N. Andrade J. D. (1974). The foreign body reaction: A chronic inflammatory response. J. Biomed. Mat. Res. 8 (5), 199–211. 10.1002/jbm.820080503 4609985

[B12] Dereli C. G. Akcan G. Can M. E. Akdere Özge E. Çaylı S. Şimşek G. (2020). Surgical and immunohistochemical outcomes of scleral reconstruction with autogenic, allogenic and xenogenic grafts: An experimental rabbit model. Curr. Eye Res. 45 (12), 1572–1582. 10.1080/02713683.2020.1764976 32366164

[B13] Fang Y. Yokoi T. Nagaoka N. Shinohara K. Onishi Y. Ishida T. (2018). Progression of myopic maculopathy during 18-year follow-up. Ophthalmology 125 (6), 863–877. 10.1016/j.ophtha.2017.12.005 29371011

[B14] Farokhi M. Mottaghitalab F. Samani S. Shokrgozar M. A. Kundu S. C. Reis R. L. (2018). Silk fibroin/hydroxyapatite composites for bone tissue engineering. Biotechnol. Adv. 36 (1), 68–91. 10.1016/j.biotechadv.2017.10.001 28993220

[B15] Gambari L. Amore E. Raggio R. Bonani W. Barone M. Lisignoli G. (2019). Hydrogen sulfide-releasing silk fibroin scaffold for bone tissue engineering. Mat. Sci. Eng. C 102, 471–482. 10.1016/j.msec.2019.04.039 31147018

[B16] Gavrieli Y. Sherman Y. Ben-Sasson S. A. (1992). Identification of programmed cell death *in situ* via specific labeling of nuclear DNA fragmentation. J. Cell Biol. 119 (3), 493–501. 10.1083/jcb.119.3.493 1400587 PMC2289665

[B17] Gentle A. Liu Y. Martin J. E. Conti G. L. McBrien N. A. (2003). Collagen gene expression and the altered accumulation of scleral collagen during the development of high myopia. J. Biol. Chem. 278, 16587–16594. 10.1074/jbc.m300970200 12606541

[B18] Hayashi K. Ohno-Matsui K. Shimada N. Moriyama M. Kojima A. Hayashi W. (2010). Long-term pattern of progression of myopic maculopathy: A natural history study. Ophthalmology 117 (8), 1595–1611. 10.1016/j.ophtha.2009.11.003 20207005

[B19] Hofmann S. Hagenmüller H. Koch A. M. Müller R. Vunjak-Novakovic G. Kaplan D. L. (2007). Control of *in vitro* tissue-engineered bone-like structures using human mesenchymal stem cells and porous silk scaffolds. Biomaterials 28 (6), 1152–1162. 10.1016/j.biomaterials.2006.10.019 17092555

[B20] Hong H. Seo Y. B. Kim D. Y. Lee J. S. Lee Y. J. Lee H. (2020). Digital light processing 3D printed silk fibroin hydrogel for cartilage tissue engineering. Biomaterials 232, 119679. 10.1016/j.biomaterials.2019.119679 31865191

[B21] Horan R. L. Antle K. Collette A. L. Wang Y. Huang J. Moreau J. E. (2005). *In vitro* degradation of silk fibroin. Biomaterials 26 (17), 3385–3393. 10.1016/j.biomaterials.2004.09.020 15621227

[B22] Huang W. Duan A. Qi Y. (2019). Posterior scleral reinforcement to prevent progression of high myopia. Asia-Pac. J. Ophthalmol. 8 (5), 366–370. 10.1097/apo.0000000000000257 PMC6784774 31513040

[B23] Jacob-LaBarre J. T. Assouline M. Byrd T. McDonald M. (1994). Synthetic scleral reinforcement materials: I. Development and *in vivo* tissue biocompatibility response. J. Biomed. Mat. Res. 28 (6), 699–712. 10.1002/jbm.820280607 8071381

[B24] Kapoor S. Kundu S. C. (2016). Silk protein-based hydrogels: Promising advanced materials for biomedical applications. Acta Biomater. 31, 17–32. 10.1016/j.actbio.2015.11.034 26602821

[B25] Kassem R. R. Abdel-Hamid M. A. Khodeir M. M. (2011). Effect of lyophilized amniotic membrane on the development of adhesions and fibrosis after extraocular muscle surgery in rabbits. Curr. Eye Res. 36 (11), 1020–1027. 10.3109/02713683.2011.601842 21942278

[B26] Keeley F. W. Morin J. D. Vesely S. (1984). Characterization of collagen from normal human sclera. Exp. Eye Res. 39 (5), 533–542. 10.1016/0014-4835(84)90053-8 6519194

[B27] Kundu B. Rajkhowa R. Kundu S. C. Wang X. (2013). Silk fibroin biomaterials for tissue regenerations. Adv. Drug Deliv. Rev. 65 (4), 457–470. 10.1016/j.addr.2012.09.043 23137786

[B28] Li H. Wang Y. Sun X. Tian W. Xu J. Wang J. (2019). Steady-state behavior and endothelialization of a silk-based small-caliber scaffold *in vivo*. Transplantation. Polymers 11 (8), 1303. 10.3390/polym11081303 31382650 PMC6723494

[B29] Lovett M. Cannizzaro C. Daheron L. Messmer B. Vunjak-Novakovic G. Kaplan D. L. (2007). Silk fibroin microtubes for blood vessel engineering. Biomaterials 28 (35), 5271–5279. 10.1016/j.biomaterials.2007.08.008 17727944 PMC2695960

[B30] Lu S. Wang P. Zhang F. Zhou X. Zuo B. You X. (2015). A novel silk fibroin nanofibrous membrane for guided bone regeneration: A study in rat calvarial defects. Am. J. Transl. Res. 7 (11), 2244–2253. 26807172 PMC4697704

[B31] Maity B. Samanta S. Sarkar S. Alam S. Govindaraju T. (2020). Injectable silk fibroin-based hydrogel for sustained insulin delivery in diabetic rats. ACS Appl. Bio Mat. 3 (6), 3544–3552. 10.1021/acsabm.0c00152 35025224

[B32] McBrien N. A. Gentle A. (2003). Role of the sclera in the development and pathological complications of myopia. Prog. Retin. Eye Res. 22 (3), 307–338. 10.1016/s1350-9462(02)00063-0 12852489

[B33] Meinel L. Hofmann S. Karageorgiou V. Kirker-Head C. McCool J. Gronowicz G. (2005). The inflammatory responses to silk films *in vitro* and *in vivo* . Biomaterials 26 (2), 147–155. 10.1016/j.biomaterials.2004.02.047 15207461

[B34] Minoura N. Tsukada M. Nagura M. (1990). Physico-chemical properties of silk fibroin membrane as a biomaterial. Biomaterials 11 (6), 430–434. 10.1016/0142-9612(90)90100-5 2207234

[B35] Mottaghitalab F. Hosseinkhani H. Shokrgozar M. A. Mao C. Yang M. Farokhi M. (2015). Silk as a potential candidate for bone tissue engineering. J. Control. Release 215, 112–128. 10.1016/j.jconrel.2015.07.031 26254197

[B36] Ni Y. Jiang Y. Wang K. Shao Z. Chen X. Sun S. (2019). Chondrocytes cultured in silk-based biomaterials maintain function and cell morphology. Int. J. Artif. Organs 42 (1), 31–41. 10.1177/0391398818806156 30376753

[B37] Ni Y. Jiang Y. Wen J. Shao Z. Chen X. Sun S. (2014). Construction of a functional silk-based biomaterial complex with immortalized chondrocytes *in vivo* . J. Biomed. Mat. Res. Part A 102 (4), 1071–1078. 10.1002/jbm.a.34763 23625883

[B38] Ohno-Matsui K. Lai T. Y. Y. Lai C.-C. Cheung C. M. G. (2016). Updates of pathologic myopia. Prog. Retin. Eye Res. 52, 156–187. 10.1016/j.preteyeres.2015.12.001 26769165

[B39] Rada J. A. Achen V. R. Perry C. A. Fox P. W. (1997). Proteoglycans in the human sclera. Evidence for the presence of aggrecan. Invest. Ophthalmol. Vis. Sci. 38 (9), 1740–1751. 9286262

[B40] Rada J. A. S. Shelton S. Norton T. T. (2006). The sclera and myopia. Exp. Eye Res. 82 (2), 185–200. 10.1016/j.exer.2005.08.009 16202407

[B41] Saw S. M. Gazzard G. Au Eong K.-G. Tan D. T. H. (2002). Myopia: Attempts to arrest progression. Br. J. .Ophthalmol. 86 (11), 1306–1311. 10.1136/bjo.86.11.1306 12386095 PMC1771373

[B42] Schepens C. L. Acosta F. (1991). Scleral implants: An historical perspective. Surv. Ophthalmol. 35 (6), 447–453. 10.1016/0039-6257(91)90108-r 1882323

[B43] Shevelev M. M. (1930). Operation against high myopia and sclera with aid of the transplantation of fascia lata on thinned sclera. Russ. Oftalmol. J. 11, 107–110.

[B44] Snyder A. A. Thompson F. B. (1972). A simplified technique for surgical treatment of degenerative myopia. Am. J. Ophthalmol. 74 (2), 273–277. 10.1016/0002-9394(72)90544-2 5054236

[B45] Thompson F. B. (1978). A simplified scleral reinforcement technique. Am. J. Ophthalmol. 86 (6), 782–790. 10.1016/0002-9394(78)90121-6 736075

[B46] Wang H. Zhang Y. (2015). Processing silk hydrogel and its applications in biomedical materials. Biotechnol. Prog. 31 (3), 630–640. 10.1002/btpr.2058 25740113

[B47] Wang X. Zhang X. Castellot J. Herman I. Iafrati M. Kaplan D. L. (2008). Controlled release from multilayer silk biomaterial coatings to modulate vascular cell responses. Biomaterials 29 (7), 894–903. 10.1016/j.biomaterials.2007.10.055 18048096 PMC2693052

[B48] Wang Y. Blasioli D. J. Kim H.-J. Kim H. S. Kaplan D. L. (2006). Cartilage tissue engineering with silk scaffolds and human articular chondrocytes. Biomaterials 27 (25), 4434–4442. 10.1016/j.biomaterials.2006.03.050 16677707

[B49] Wang Y. Rudym D. D. Walsh A. Abrahamsen L. Kim H.-J. Kim H. S. (2008). *In vivo* degradation of three-dimensional silk fibroin scaffolds. Biomaterials 29 (24-25), 3415–3428. 10.1016/j.biomaterials.2008.05.002 18502501 PMC3206261

[B50] Xue A. Bao F. Zheng L. Wang Q. Cheng L. Qu J. (2014). Posterior scleral reinforcement on progressive high myopic young patients. Optom. Vis. Sci. 91 (4), 412–418. 10.1097/opx.0000000000000201 24509544

[B51] Yan L.-P. Silva-Correia J. Oliveira M. B. Vilela C. Pereira H. Sousa R. A. (2015). Bilayered silk/silk-nanoCaP scaffolds for osteochondral tissue engineering: *In vitro* and *in vivo* assessment of biological performance. Acta Biomater. 12, 227–241. 10.1016/j.actbio.2014.10.021 25449920

[B52] Yan Z. Chen W. Jin W. Sun Y. Cai J. Gu K. (2021). An interference screw made using a silk fibroin-based bulk material with high content of hydroxyapatite for anterior cruciate ligament reconstruction in a rabbit model. J. Mat. Chem. B 9 (26), 5352–5364. 10.1039/d1tb01006a 34152356

[B53] Yan Z. Wang C. Chen W. Song X. (2010). Biomechanical considerations: Evaluating scleral reinforcement materials for pathological myopia. Can. J. Ophthalmol. 45 (3), 252–255. 10.3129/i09-279 20436547

[B54] Zhang X. Baughman C. B. Kaplan D. L. (2008). *In vitro* evaluation of electrospun silk fibroin scaffolds for vascular cell growth. Biomaterials 29 (14), 2217–2227. 10.1016/j.biomaterials.2008.01.022 18279952 PMC2698960

[B55] Zheng H. Zuo B. (2021). Functional silk fibroin hydrogels: Preparation, properties and applications. J. Mat. Chem. B 9 (5), 1238–1258. 10.1039/d0tb02099k 33406183

[B56] Zhou C. Z. Confalonieri F. Jacquet M. Perasso R. Li Z. G. Janin J. (2001). Silk fibroin: Structural implications of a remarkable amino acid sequence. Proteins 44 (2), 119–122. 10.1002/prot.1078 11391774

