# Supplementary material for: Evaluation of new robust silk fibroin hydrogels for posterior scleral reinforcement in rabbits
Source: Front Bioeng Biotechnol. 2023 Jun 14;11:1211688. doi: 10.3389/fbioe.2023.1211688 (PMC10300450; doi:10.3389/fbioe.2023.1211688)
Supplement: Supplementary file 1 [file Table1.DOCX]

**Table S1** Parameters for histopathologic evaluation

| Parameters | Values |
| --- | --- |
| Inflammation  Vascularization | 0 = no inflammation  1 = a few lymphocytes and plasma cells  2 = mild inflammatory infiltrate composed of lymphocytes, plasma cells and polymorphonuclear leucocytes (low number of neutrophils)  3 = grade two plus neutrophils (high number of neutrophils)  4 = high concentrations of lymphocytes, plasma cells, polymorphnuclear leucocytes and histiocytes  0 = avascularity  1 = some avascularity  2 = normal vascularity  3 = mildly increased vascularity suggestive of ongoing inflammation  4 = severely increased vascularity |
