# Supplementary figures and images for: Evaluation of new robust silk fibroin hydrogels for posterior scleral reinforcement in rabbits
Source: Front Bioeng Biotechnol. 2023 Jun 14;11:1211688. doi: 10.3389/fbioe.2023.1211688 (PMC10300450; doi:10.3389/fbioe.2023.1211688)

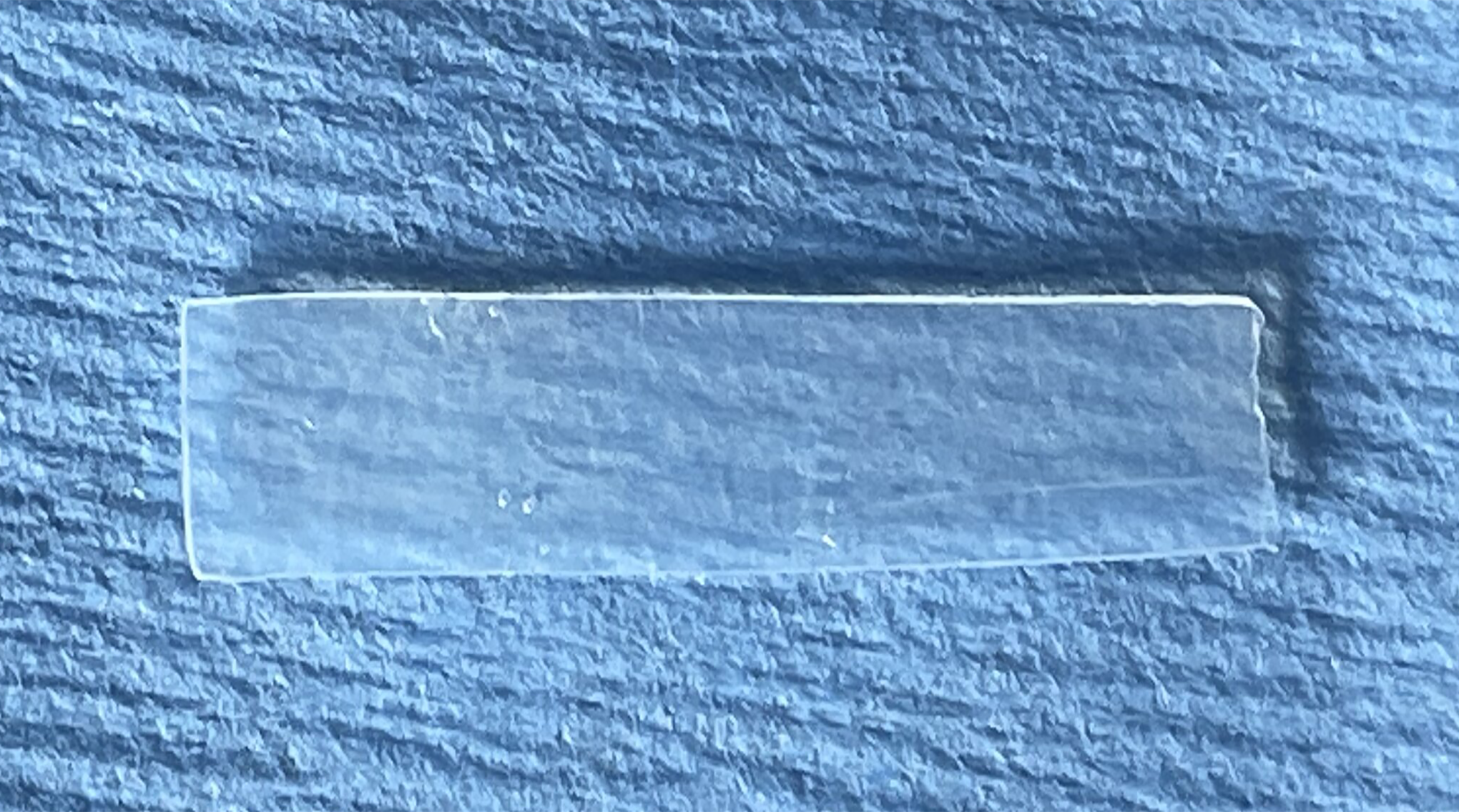

Supplement: Supplementary file 2 [file Image1.TIF]
